# Maternal *Chlamydia trachomatis* Infections and Preterm Births in a University Hospital in Vitoria, Brazil

**DOI:** 10.1371/journal.pone.0141367

**Published:** 2015-10-27

**Authors:** Renylena Schmidt, Renan Rosetti Muniz, Elizandra Cola, Dulce Stauffert, Mariangela Freitas Silveira, Angelica E. Miranda

**Affiliations:** 1 Programa de Pós-Graduação em Doenças Infecciosas, Universidade Federal do Espirito Santo, Vitoria, Brasil; 2 Departamento Materno Infantil, Faculdade de Medicina, Universidade Federal de Pelotas, Pelotas, Brasil; 3 Programa de Pós-Graduação em Epidemiologia, Universidade Federal de Pelotas, Pelotas, Brasil; John Hunter Hospital, AUSTRALIA

## Abstract

**Background:**

Preterm birth (PTB) is a major determinant of neonatal morbimortality with adverse consequences for health. The causes are multifactorial, with intrauterine infection probably explaining most of these outcomes. It is believed that infection with *Chlamydia trachomatis* (CT) is also involved in PTB and premature rupture of membranes.

**Objetives:**

To evaluate the prevalence of and associated factors for CT among cases of PTB attended at a University Hospital in Vitoria, Brazil.

**Methods:**

A cross-sectional study performed among parturient who had preterm birth from June 2012 to August 2013 in Vitoria, Brazil. Participants answered a questionnaire including demographic, behavioral, and clinical data. A sample of urine was collected and screened for CT using polymerase chain reaction. Chi-square tests were used for proportion differences and Student’s-t tests and variance analysis were used for testing differences between mean values. Odds ratio was used as a measure of association with a 95% confidence interval.

**Results:**

The prevalence of PTB during the period of the study was 26% and the prevalence of CT among them was 13.9%. A total of 31.6% pregnant women were younger than 25 years old and women infected by CT were even younger than women not infected by CT (p = 0.022). Most of them (76.2%) were married or had a living partner, and CT infection was more frequent among the single ones (p = 0.018); 16.7% of women reported their first sexual intercourse under 14 years old. The causes of prematurity were maternal-fetal in 40.9%; rupture of the membranes in 29.7% and premature labor in 29.4%. In multivariate analysis, being married was a protective factor for infection [OR = 0.48 (95%CI:0.24–0.97)]. None of the other characteristics were associated with CT infection.

**Conclusions:**

This study shows a high prevalence of CT infection among parturient who have preterm birth. This high prevalence highlight the need for defining screening strategies focused on young pregnant women in Brazil.

## Introduction

Among the neonatal causes of infant mortality, 61.4% are associated with preterm birth (PTB), such as hypoxia, neonatal infection, respiratory distress syndrome, and other respiratory problems. Thus, preterm birth (PTB) plays an important role in relation to infant mortality and adequate control; and its management are potentially effective interventions for reducing this mortality [1] [2]. The complications of PTB cause approximately 70% of neonatal deaths and nearly half of all long-term neurological morbidity [3] [4]. The impact on the individual child and their family, combined with the associated socioeconomic costs of health care, make PTB a major public health concern [5].

There is evidence that sexually transmitted infections (STI) can impact the development of preterm labor, as well as other complications of pregnancy, such as miscarriage, stillbirth, and preeclampsia [6]. Serologic evidence of acute infection with *Chlamydia trachomatis* (CT), as measured by IgM titers, has been associated with preterm labor, while IgG antibody was associated with stillbirth [7][8], and elevated IgM antibodies to CT were reported to be associated with recurrent spontaneous miscarriage [9]. This suggests that preexisting immunity may alter the impact of CT infection on pregnancy outcome.

Epidemiologic data suggests a link between CT infection and adverse pregnancy outcomes, in addition to its association with infertility [7][8]. Transmission of CT to infants during birth, particularly in the setting of premature rupture of membranes, is a known risk factor for the development of conjunctivitis and pneumonia [10]. Many studies have also shown an association between first trimester infection with CT and miscarriage [8][11]. The role of CT later in pregnancy is less clear, although serologic evidence of acute infection with CT during pregnancy has also been associated with stillbirth and preterm delivery [7], and at least one study from the Netherlands suggested that 14.9% of PTB before 32 weeks and 7.4% of PTB before 35 weeks gestation were attributable to CT infection [12]. A previous Brazilian study performed to determine the prevalence and risk behaviors for CT in young parturient women found a rate of 9.8% but did not found association between CT and preterm birth [13].

Official data from the Brazilian Ministry of Health showed a prevalence of preterm birth in Brazil of 6.5% in 2009 [14]. A systematic review conducted in 2008 found a prevalence of preterm labor ranging from 3.4% to 15.0% in the South and Southeast regions between 1978 and 2004, and from 3.8% to 10.2% in the Northeast region between 1984 and 1998. [15]. In another study performed among young women attending public hospitals in Brazil was described a prevalence of 21.7% for preterm labor among women from 15 to 24 years old. The highest proportion of PTB (36.1%) was found in the North region and the lowest (6.9%) was found in the South region of the country [16]. A multicentric study showed a prevalence of 12.3% of preterm births in a sample of selected tertiary referral Brazilian maternities [17].

PTB can be the result of preterm labor with intact membranes; preterm with rupture of membranes, and medically indicated [6]. The goal of this study was to evaluate the prevalence of CT in pregnant women who spontaneously delivery a preterm baby at a University Hospital in Vitoria, Brazil. The results will be used in the elaboration of public health policies both to recommend preventive actions and to identify indicators for monitoring preterm labor prevention strategies.

## Methods

This is a cross-sectional study performed among parturient women attending a public University hospital in Vitória, Brazil. Parturient women attending the maternity unit and having a spontaneous preterm labor in 2013 were invited to take part in the study.

All pregnant women, who have preterm birth (delivery occurred at gestational ages between 22 weeks (or> 500g) and 36 weeks and 6 days in a University Hospital in Vitória, from June 2012 to August 2013 were invited to take part in the study. Gestational age was calculated by Nagele rule for women with date of the last reported menstrual period, and confirmed by the results of transvaginal ultrasound exams. Pregnancies poorly dated that Capurro score of newborn had not confirmed prematurity were excluded from the study.

Each participant was interviewed face-to-face by a trained health professional for collection of socio-demographic data (age, race/color, schooling, marital status and family income); clinical data (gestational age, number of pregnancies, number of childbirths, number of miscarriages/abortions, antenatal examinations performed); sexual data (age at first sexual intercourse, prior history of STI, gynecological complaints, number of sex partners in the last year and since their first sexual intercourse); STI/HIV risk behavior (drug use, sex in exchange for money/goods and information about sex partners regarding a history of blood transfusions, injecting drug use, bisexual practices and history of imprisonment).

A 20 ml urine sample was collected, from the first amount of urine flow, with the recommendations of no prior genital cleansing and a minimum period of two hours without urinating prior to sample collection. Samples were analyzed by PCR using the BD ProbeTecTM CT / GC Amplified DNA Assay Collection Kit for Endocervical Specimens (Franklin Lakes, NJ, USA) for qualitative in vitro detection of CT, as per the manufacturer’s instructions at the Molecular Biology Laboratory of the Federal University of Pelotas, Rio Grande do Sul, Brazil.

Data were analyzed using the SPSS–data entry statistical program (Statistical Package for the Social Sciences) version 17.0. A preliminary analysis was performed using exploratory techniques on the data, to check the distribution patterns and trends of the principal variables. Bivariate analysis was then performed to check for the presence of association between the variables. Chi-square (χ2) tests were used for proportion differences and Student’s-t tests and variance analysis were used for testing differences between mean values. Univariate and multivariate odds ratios (ORs) (adjusting for potential confounders) and 95% confidence intervals (CIs) were reported. Variables that were significant at P<0.20 in bivariate analysis, and known confounders (e.g., age and education), were considered in the multivariate analysis using a stepwise multiple logistic regression model.

This project was submitted to and approved by the Research Ethics Committee (#131107/2012) of the University Hospital of the Federal University of Espírito Santo. All selected women were invited to take part voluntarily in the study and those who accepted signed a written consent form. Those who were diagnosed as being infected by CT received treatment in accordance with the Brazilian Ministry of Health guidelines.

## Results

During the study period, 1,452 deliveries occurred in the university hospital. Of these, 1,074 were at term and 378 preterm. Among them 323 women filled the inclusion criteria, accepted to participate and were included in the study. The prevalence of CT among the preterm births cases was 13.9% (45 cases).

Table 1 describes demographic and behavioral characteristics, 31.6% were 24 years old or younger and women infected by CT were even younger than women not infected by CT [OR = 2.0(IC95%CI:1.1–3.8), p = 0.022].

**Table 1:** Demographic and behavior characteristics reported by parturient women delivering preterm babies in Vitoria, 2013 by *Chlamydia trachomatis* (n = 323).

Variable	Total	CT +	CT-	
	N (%)	N (%)	N (%)	p value
**Age (years)**				**0.032**
Up to 24	**146 (45.2)**	**27 (60.0)**	**119 (42.8)**	
More than 24	**177 (54.8)**	**18 (40.0)**	**159 (57.2)**	
**Education (years)**				**0.665**
< = 8	**146 (45.2)**	**19 (42.2)**	**127 (45.7)**	
>8	**177 (54.8)**	**26 (57.8)**	**151 (54.3)**	
**Marital status**				**0.018**
Married/living together	**246 (76.2)**	**28 (62.2)**	**218 (78.4)**	
Single	**77 (23.8)**	**17 (37.8)**	**60 (21.6)**	
**First sex intercourse**				**0.846**
< = 13 years	**54 (16.7)**	**08 (17.8)**	**46 (16.5)**	
14–17 years	**186 (57.6)**	**27 (60.0)**	**159 (57.2)**	
> = 18 years	**83 (25.7)**	**10 (22.2)**	**73 (26.3)**	
**Number of partners (life)**				**0.413**
Only one	**106 (32.8)**	**17 (37.8)**	**89 (32.0)**	
2–5	**168 (52.0)**	**24 (53.3)**	**144 (51.8)**	
6–10	**49 (15.2)**	**04 (8.9)**	**45 (51.8)**	
**Tobacco in pregnancy**				**0.633**
Yes	**43 (13.3)**	**07 (15.6)**	**36 (12.9)**	
No	**280 (86.7)**	**38 (84.4)**	**242 (87.1)**	
**Alcohol abuse in pregnancy**				**0.622**
Yes	**29 (9.0)**	**04 (8.9)**	**25 (9.0)**	
No	**294 (91.0)**	**41 (91.1)**	**253 (91.0)**	
**Illicit drug abuse**				**0.417**
Yes	**10 (3.1)**	**02 (4.4)**	**08 (2.9)**	
No	**313 (96.9)**	**43 (95.6)**	**270 (97.1)**	
**Antenatal care**				**0.404**
Yes	**250 (77.4)**	**37 (82.2)**	**213 (76.6)**	
No	**73 (22.6)**	**08 (17.8)**	**65 (23.4)**	

A total of 76.2% were married/living together, but CT was more frequent among the single ones [OR = 2.2 (IC95%CI: 1.1–4.3), p = 0.018]; 16.7% of women had their first sexual activity under 14 years old, and 77.4% had at least 4 prenatal visits, as recommended by the World Health Organization. Antenatal care was not a protective factor for CT infection (p = 0.404).

Regarding clinical characteristics, 61.0% had premature rupture of membranes (PROM) and 41.8% presented spontaneous labor. The PROM was more frequent among women infected by CT but the difference was not significant [OR = 1.4 (IC95%CI:0.6–3.1, p = 0.404)]. The causes of prematurity were maternal-fetal in 40.9%, rupture of the membranes in 29.7% and premature labor in 29.4%. Vaginal delivery frequency was similar between women infected by CT and women with a negative test result (51.1% vs. 42.4%), OR = 1.4 (95%CI: 0.8–2.6). None of these characteristics were associated with CT infection (Table 2).

**Table 2:** Clinical characteristics reported by parturient women delivering preterm babies in Vitoria, 2013 by Chlamydia trachomatis (n = 323).

Variable	Total	CT +	CT-	
	N (%)	N (%)	N (%)	p value
**Any infection in pregnancy**				0.694
Yes	123 (38.1)	16 (35.6)	107 (38.5)	
No	200 (61.9)	29 (64.4)	171 (61.5)	
**Rupture of membranes**				0.158
Spontaneous	199 (61.6)	32 (71.1)	167 (60.1)	
Artificial	124 (38.4)	13 (28.9)	111 (39.9)	
**Syphilis**				0.531
Yes	11 (3.4)	01 (2.2)	10 (3.6)	
No	312 (96.6)	44 (97.8)	268 (96.4)	
**Streptococcus B**				0.597
Yes	06 (1.9)	01 (2.2)	05 (1.8)	
No	317 (98.1)	44 (97.8)	273 (98.2)	
**Urinary infection**				0.937
Yes	106 (33.8)	15 (33.3)	91 (32.7)	
No	217 (67.2)	30 (66.7)	187 (67.3)	
**Vaginal discharge**				0.606
Yes	14 (4.3)	02 (4.4)	12 (4.3)	
No	309 (95.7)	43 (95.6)	266 (95.7)	
**Labor**				0.154
Interruption by C-section	139 (43.0)	14 (31.1)	125 (45.0)	
Spontaneous	135 (41.8)	21 (46.7)	114 (41.0)	
Induced after PROM	49 (15.2)	10 (22.2)	39 (14.0)	
**Delivery**				0.277
Vaginal	141 (43.7)	23 (51.1)	118 (42.4)	
C- Section	182 (56.3)	22 (48.9)	160 (57.6)	
**Birth weight (grams)**				0.575
< = 1500	73 (22.9)	13 (28.9)	60 (21.9)	
1501–2499	150 (47.0)	19 (42.2)	131 (47.8)	
> = 2500	96 (30.1)	13 (28.9)	83 (30.3)	
**Prematurity causes**				0.188
Maternal-fetal	132 (40.9)	13 (28.9)	119 (42.8)	
Labor	95 (29.4)	15 (33.3)	80 (28.8)	
Rupture of membranes	96 (29.7)	17 (37.8)	79 (28.4)	
**Gestational week**				0.642
Up to 32 weeks	77 (25.7)	12 (28.6)	65 (25.2)	
More than 32 weeks	223 (74.3)	30 (71.4)	193 (74.8)	
**Apgar score at 5th minute**				0.274
Up to 6	18 (6.1)	1 (2.4)	17 (6.7)	
7 or more	275 (93.9)	40 (96.6)	235 (93.3)	
**Neonatal care**				0.179
Low risk	74 (23.3)	14 (31.1)	60 (22.0)	
High risk	244 (76.7)	31 (68.9)	213 (78.0)	

In the final model of multivariate analysis, the only variable that remained inversely associated with infection by *Chlamydia trachomatis* was marital status. Being married was a protective factor for infection [OR = 0.48 (95% CI: 0.24–0.97)].

## Discussion

This study found high rates of CT infection and of PTB in a University Hospital. These finding are in agreement to previous studies that approach the prevalence of CT in pregnant women [12][18][19] and PTB [15][16][17] in Brazil. CT infection is high prevalent in young pregnant women and early detection and eradication of this STI without recurrent/persistent infection during pregnancy could be an intervention to improve the quality of care to these women and secondly it could reduce the risk of preterm birth [20].

The high prevalence of CT infection among the preterm births cases in our hospital highlights the importance of routine screening in antenatal care in our region. It is difficult to determine the cause of PTB in Brazil because the multifactorial causes including social problems; therefore earlier CT screening and treatment during pregnancy could help on its control. Available evidence suggests that when interventions in health services are effectively implemented, it can improve the quality of care and, consequently, the sexual and reproductive health [21].

The prevalence of PTB in this study was high (21.4%). This value is in agreement with data obtained in the study of Miranda et al in young pregnant women in Brazil [16], but higher than expected when compared with other data from Brazil [14][17]. A systematic review conducted in the country in 2008 found a prevalence of preterm birth ranging from 3.4% to 15.0% in the South and Southeast regions between 1978 and 2004, and 3.8% to 10.2% in Northeast between from 1984 and 1998 [15]. The hospital where we conducted the study is a reference hospital for complicated births, which can explain the higher rate of preterm births.

The present study was descriptive and only focused on preterm births and could not evaluate the association between CT and preterm birth. The possibility of response bias cannot be ruled out due to the general tendency to give socially acceptable answers. Behavior, clinical and laboratory data were checked in medical records to confirm the information and reduce bias.

The association between CT infection and preterm birth is not well establish in the literature. The CT infection in pregnancy and childbirth can trigger or promote premature labor, premature rupture of membranes, low birth weight and fetal death by virtue of its ability to chronically infect placental tissue and induce low-grade inflammation, leading to placental dysfunction, growth restriction of the fetus, and preterm birth [7][8]. It may also impact on the fetus leading to lung and eye infections [6][7].

There are published studies describing the association between CT infection and preterm birth [15][22][23]. A population-based cohort study conducted in Netherlands showed more than twice the risk of *Chlamydia* infection for preterm births below 35 weeks [12] and a second study showed an association between *chlamydia* infection and histological signs of placental inflammation in preterm births with gestational age less than 32 weeks [24]. Other studies did not find an association between preterm birth and CT, [16][22][25]. Asymptomatic women, mostly, are rarely investigated for possible risk behaviors for STI. This shows that the guidelines for early diagnosis and treatment of STI, including sexual partnerships, aiming to break the chain of transmission of these diseases are little known or assumed by health professionals.

## Conclusions

This study showed a high prevalence of CT infection among parturient who have preterm birth. This high prevalence highlights the need for defining screening strategies focused on young pregnant women in Brazil. These data also indicate the need to strengthen services for caring pregnant women at risk of preterm birth. Health programs should be aware of the need of controlling preterm birth risk factors as STI in populations at higher risk and vulnerability.

## References

[1] Lawn JE, Wilczynska-Ketende K, Cousens SN. Estimating the causes of 4 million neonatal deaths in the year 2000. Int J Epidemiol. 2006; 35(3):706–18. 16556647 10.1093/ije/dyl043

[2] Victora CG, Aquino EM, do Carmo Leal M, Monteiro CA, Barros FC, Szwarcwald CL. Maternal and child health in Brazil: progress and challenges. Lancet 2011; 28;377(9780):1863–76. 10.1016/S0140-6736(11)60138-4 21561656

[3] McCormick MC. The contribution of low birth weight to infant mortality and childhood morbidity. N Engl J Med. 1985; 312:82–90. 3880598 10.1056/NEJM198501103120204

[4] Mathews TJ, MacDorman MF. Infant mortality statistics from the 2006 period linked birth/infant death data set. Natl Vital Stat Rep. 2010; 58:1–31. 20815136

[5] Beck S, Wojdyla D, Say L, Betran AP, Merialdi M, Requejo JH et al The worldwide incidence of preterm birth: a systematic review of maternal mortality and morbidity. Bull World Health Organ. 2010; 88(1):31–8. 10.2471/BLT.08.062554 20428351 PMC2802437

[6] Goldenberg RL, Culhane JF, Iams JD, Romero R. Epidemiology and causes of preterm birth. Lancet. 2008; 371(9606):75–84. 10.1016/S0140-6736(08)60074-4 18177778 PMC7134569

[7] Gencay M, Koskiniemi M, Ammala P, Fellman V, Narvanen A, Wahlstrom T, et al Chlamydia trachomatis seropositivity is associated both with stillbirth and preterm delivery. APMIS 2000; 108:584–588. 11110046 10.1034/j.1600-0463.2000.d01-101.x

[8] Gencay M, Koskiniemi M, Fellman V, Ammala P, Vaheri A, Puolakkainen M. Chlamydia trachomatis infection in mothers with preterm delivery and in their newborn infants. APMIS 2001; 109:636–640. 11878718 10.1034/j.1600-0463.2001.d01-186.x

[9] Kishore J, Agarwal J, Agrawal S, Ayyagari A . Seroanalysis of Chlamydia trachomatis and S-TORCH agents in women with recurrent spontaneous abortions. Indian J Pathol Microbiol 2003;46:684–687. 15025382

[10] Baud D, Goy G, Jaton K, Osterheld MC, Blumer S, Borel N, et al Role of Chlamydia trachomatis in miscarriage. Emerg Infect Dis. 2011;17(9):1630–5. 10.3201/eid1709.100865 21888787 PMC3322049

[11] Baud D, Regan L, Greub G. Emerging role of Chlamydia and Chlamydia-like organisms in adverse pregnancy outcomes. Curr Opin Infect Dis. 2008; 21:70–6 10.1097/QCO.0b013e3282f3e6a5 18192789

[12] Rours GI, de Krijger RR, Ott A, Willemse HF, de Groot R. Chlamydia trachomatis and placental inflammation in early preterm delivery. Eur J Epidemiol. 2011; 26(5):421–8. 10.1007/s10654-011-9569-2 21431838 PMC3109244

[13] Pinto VM, Szwarcwald CL, Baroni C, Stringari LL, Inocêncio LA, Miranda AE. Chlamydia trachomatis prevalence and risk behaviors in parturient women aged 15 to 24 in Brazil. Sex Transm Dis. 2011;38(10):957–61. 10.1097/OLQ.0b013e31822037fc 21934572

[14] Brasil. Ministério da Saúde, Departamento de Informática do Sistema Único de Saúde (BR). Informações de saúde: nascidos vivos. Notas técnicas [Internet]. Rio de Janeiro: MS/ DATASUS. Available from: http://tabnet.datasus.gov.br/cgi/sinasc/nvdescr.htm Accessed 20 May 2015.

[15] Silveira MF, Santos IS, Barros AJ, Matijasevich A, Barros FC, Victora CG. Increase in preterm births in Brazil: review of population-based studies. Rev Saude Publica 2008; 42(5):957–64. 18833394 10.1590/s0034-89102008000500023

[16] Miranda AE, Pinto VM, Szwarcwald CL, Golub ET. Prevalence and correlates of preterm labor among young parturient women attending public hospitals in Brazil. Rev Panam Salud Publica. 2012; 32(5):330–4. 23338689 10.1590/s1020-49892012001100002

[17] Passini R Jr, Cecatti JG, Lajos GJ, Tedesco RP, Nomura ML, Dias TZ, et al Brazilian Multicentre Study on Preterm Birth study group. Brazilian multicentre study on preterm birth (EMIP): prevalence and factors associated with spontaneous preterm birth. PLoS One. 2014 10 9; 9(10):e109069 10.1371/journal.pone.0109069 eCollection 2014. 25299699 PMC4192080

[18] Jalil EM, Pinto VM, Benzaken AS, Ribeiro D, Oliveira EC, Garcia EG, et al Prevalence of Chlamydia and Neisseria gonorrhoeae infections in pregnant women in six Brazilian cities. Rev Bras Ginecol Obstet. 2008; 30(12):614–9. 19219343 10.1590/s0100-72032008001200005

[19] Borborema-Alfaia AP, Freitas NS, Astolfi Filho S, Borborema-Santos CM. Chlamydia trachomatis infection in a sample of northern Brazilian pregnant women: prevalence and prenatal importance. Braz J Infect Dis. 2013; 17(5):545–50. 10.1016/j.bjid.2013.01.014 23831212 PMC9425139

[20] Folger AT. Maternal Chlamydia trachomatis infections and preterm birth: the impact of early detection and eradication during pregnancy. Matern Child Health J. 2014; 18(8):1795–802. 10.1007/s10995-013-1423-6 24337865

[21] NgBE, ButlerLM, HorvathT, RutherfordGW. Population-based biomedical sexually transmitted infection control interventions for reducing HIV infection. Cochrane Database Syst Rev. 2011 3 16; (3):CD001220. 10.1002/14651858.CD001220.pub3 21412869

[22] Andrews WW, Goldenberg RL, Mercer B, Iams J, Meis P, Moawad A, et al The preterm prediction study: association of second-trimester genitourinary chlamydia infection with subsequent spontaneous preterm birth. Am J Obstet Gynecol. 2000; 183(3):662–668. 10992190 10.1067/mob.2000.106556

[23] Liu B, Roberts CL, Clarke M, Jorm L, Hunt J, Ward J. Chlamydia and gonorrhoea infections and the risk of adverse obstetric outcomes: a retrospective cohort study. Sex Transm Infect. 2013; 89(8):672–8. 10.1136/sextrans-2013-051118 24005255

[24] Rours GI, Duijts L, Moll HA, Arends LR, de Groot R, Jaddoe VW, et al Chlamydia trachomatis infection during pregnancy associated with preterm delivery: a population-based prospective cohort study. Eur J Epidemiol. 2011; 26(6):493–502. 10.1007/s10654-011-9586-1 21538042 PMC3115062

[25] Blas MM, Canchihuaman FA, Alva IE, Hawes SE. Pregnancy outcomes in women infected with Chlamydia trachomatis: a population-based cohort study in Washington State. Sex Transm Infect 2007; 83:314–318. 17344249 10.1136/sti.2006.022665 PMC2598687

